# A novel m7G RNA methylation-related signature associated with MAPK signaling pathways in acute ischemic stroke

**DOI:** 10.1371/journal.pone.0352280

**Published:** 2026-06-23

**Authors:** Wei-Gang Gong, Jian-Wei Lou, Sen Yin, Fang-Pu Yu, Wei Wu

**Affiliations:** 1 Department of Neurology, Qilu Hospital, Cheeloo College of Medicine, Shandong University, Jinan, China; 2 The Key Laboratory of Cardiovascular Remodeling & Function Research, Chinese Ministry of Education, Chinese Ministry of Health & Chinese Academy of Medical Sciences, & The State & Shandong Province Joint Key Laboratory of Translational Cardiovascular Medicine, Qilu Hospital, Cheeloo College of Medicine, Shandong University, Jinan, China; AMET University, INDIA

## Abstract

**Objectives:**

Ischemic stroke represents a major global cause of disability and death. Timely recanalization of occluded vessels to salvage the ischemic penumbra is the cornerstone of acute ischemic stroke (AIS) treatment. Nevertheless, the molecular mechanisms driving the transition from salvageable penumbra to infarct core in early AIS remain poorly understood. Recent evidence highlights the role of post-transcriptional methylation, especially N7-methylguanosine (m7G) modification, in stroke pathogenesis. This study aimed to systematically characterize transcriptome-wide m7G methylation changes in the ischemic penumbra during early AIS and to explore their potential functional relevance.

**Methods:**

We performed middle cerebral artery occlusion in 8-week-old male BALB/c mice and applied MeRIP-seq to profile the transcriptome-wide m7G methylome in penumbra tissue. Bioinformatics analyses were conducted to elucidate the functional relevance of m7G-methylated transcripts.

**Results:**

Compared with controls, AIS penumbra exhibited significant alterations in m7G-modified mRNAs. Specifically, upregulated m7G modifications were notably enriched in mRNAs associated with the mitogen-activated protein kinase (MAPK) signaling pathway, whereas downregulated modifications were significantly linked to axon guidance pathways.

**Conclusions:**

This study provides the first systematic landscape of m7G methylation in AIS and m7G RNA methylation-related alterations in the MAPK signaling pathway may serve as potential therapeutic targets for stroke intervention.

## Introduction

Ischemic stroke represents a major global cause of death and long-term disability, characterized by severe neurological impairment [1]. A critical therapeutic goal in acute ischemic stroke (AIS) is the timely restoration of blood flow to salvage the at-risk ischemic penumbra (PEN). Without timely reperfusion, typically within the critical 24-h window from symptom onset, the ischemic penumbra will inevitably deteriorate into an irreversible infarct core tissue [2]. However, the duration of penumbra survival varies significantly among patients, ranging from hours to days, reflecting the high heterogeneity of AIS. Consequently, understanding the molecular and biological changes within the ischemic penumbra is essential for developing targeted strategies to protect vulnerable tissue and enhance clinical recovery.

Over the past decade, genomic sequencing has revealed recurrent mutations in genes encoding epigenetic regulators, strongly implicating dysregulated epigenetic mechanisms in the pathogenesis of ischemic stroke. Among these, aberrant RNA methylation has emerged as a significant contributing factor. While historically recognized as a component of the 5’ mRNA cap, N7-methylguanosine (m7G) modifications have also been identified at internal sites within various RNA species, including mRNAs, tRNAs, and rRNAs [3, 4]. These internal m7G marks play crucial roles in diverse aspects of RNA biology, such as RNA processing, stability, maturation, and translational efficiency [5]. The deposition of m7G is dynamically controlled by specific methyltransferases. The most extensively studied methyltransferase for internal m7G is methyltransferase-like 1 (METTL1), the mammalian counterpart of yeast Trm8 [6]. METTL1 functions in concert with its essential cofactor, WD repeat domain 4 (WDR4), the human ortholog of yeast Trm82, forming a conserved complex responsible for catalyzing this modification [7].

Despite progress in m7G research, the precise locations and functional roles of internal m7G modifications remain largely undefined. Emerging evidence suggests that internal m7G methylation is extensively linked to dysregulation of disease-associated proteins and may represent a novel epitranscriptomic mechanism underlying several neurological disorders [8]. To elucidate the involvement of m7G in ischemic stroke, we applied MeRIP-seq to generate the first transcriptome-wide m7G methylome profile of ischemic penumbra tissue. Using m7G-specific analysis and comprehensive bioinformatics approaches, we characterized mRNA methylation patterns in ischemic versus control tissues. RNA-seq was also performed to identify differentially expressed genes, followed by an integrative analysis of differentially methylated and expressed genes.

Our findings demonstrate substantial alterations in m7G-methylated genes in ischemic penumbra tissue, with both the number of methylated genes and methylation peaks significantly higher than those in controls. Notably, m7G methylation changes in numerous stroke-related genes were associated with altered mRNA expression levels. Bioinformatics analysis further indicated that differential methylation between groups could lead to varied changes in cellular functions. Collectively, this study suggests a potential association between ischemic penumbra tissue and mRNA m7G methylation, predicts functional alterations arising from these methylation differences, and may offer new insights for advancing ischemic stroke treatment.

## Materials and methods

### Study approval

All experimental protocols in this study received approval from the Ethics Committee of Qilu Hospital of Shandong University (Approval Code: KYLL-2024(KS)-493; Approval Date: 1-20-2024). Furthermore, all animal-related procedures were reviewed and specifically authorized by the Institutional Animal Care and Use Committee (IACUC) of the same institution.

### Animals

A total of 12 adult male Balb/c mice (8 weeks old, weighing 22.0–25.5 g, Beijing Vital River Laboratory Animal Technology Co., Ltd.) were used in this study. Mice were assigned unique identification numbers generated by a random number generator and allocated into experimental groups using a remainder-based randomization method. To ensure balanced group sizes, any group found to have fewer animals than required following initial allocation was supplemented with subjects from the next available remainder assignment.

Animals were housed under strictly controlled temperature and humidity conditions with continuous access to food and water. All procedures adhered to the recommendations outlined in the NIH Guide for the Care and Use of Laboratory Animals (NIH Publications No. 8023, revised 1978) and were conducted in compliance with the Animal Experimentation Guidelines of Shandong University.

### Middle cerebral artery occlusion (MCAO)

Adult mice were anesthetized using isoflurane (4% for induction in a chamber, followed by 1.5% for maintenance). Insert the Nylon Monofilament Suture (1620A1, Beijing Sinoscience Technology Co., Ltd.) through the incision and slide it through the CCA. While gently pulling the ECA down wards, navigate the filament toward the internal carotid artery (ICA). The filament is typically advanced through the ICA until it reaches the origin of the MCA. The ischemia duration is 1 hour, and euthanasia is performed 2 hours after successful modeling [9]. The mice were euthanized by an intraperitoneal injection of pentobarbital sodium (150 mg/kg, P3761 Sigma-Aldrich), and the brain was removed for further analysis, and all efforts were made to minimize suffering. Ischemic penumbra tissue was subsequently dissected from the ipsilateral hemisphere [10]. The tissue was then rinsed in pre-chilled PBS or saline to remove blood stains and debris. The target ischemic penumbra tissue was rapidly dissected on ice. Using forceps, the tissue block was picked up and directly immersed into liquid nitrogen, ensuring that the tissue was completely submerged. After freezing for several seconds until the tissue became completely hardened, the snap-frozen tissue block was removed and quickly transferred into a pre-chilled, labeled cryotube. The cryotube was then stored in a −80°C freezer for long-term preservation.

This study complies with the ARRIVE guidelines (Animal Research: Reporting of In Vivo Experiments). Inclusion criteria were a reduction in cerebral blood flow (CBF) of ≥70% as measured by laser Doppler flowmetry and a neurological behavior score of ≥1. Exclusion criteria included intraoperative death, absence of significant CBF reduction, or the presence of subarachnoid hemorrhage. No animals died during the modeling procedure. After surgery, the animals were placed in temperature-controlled cages (37 ± 0.5°C) until they regained consciousness. They were housed individually and provided with moistened chow and hydrogel for hydration. Neurological behavior and the condition of the surgical incision were monitored. Analgesic treatment was administered in accordance with the regulations on laboratory animal welfare.

### RNA extraction and fragmentation

Total RNA was extracted from mouse penumbra tissue using TRIzol reagent (Invitrogen Corporation, CA, USA) according to the manufacturer’s instructions. RNA concentration was determined using a NanoDrop ND-100 spectrophotometer (Thermo Fisher Scientific, Waltham, MA, USA). RNA purity was assessed based on the OD260/OD280 ratio, with values between 1.8 and 2.1 considered acceptable. RNA integrity and genomic DNA contamination were evaluated by denaturing agarose gel electrophoresis.

### Merip library construction and sequencing

The m7G-IP-Seq service was performed by CloudSeq Inc. (Shanghai, China). mRNA was first isolated from total RNA using oligo(dT) magnetic beads (Thermo Fisher) and subsequently subjected to immunoprecipitation with the GenSeq™ m7G-IP Kit (GenSeq Inc., China) following the manufacturer’s protocol. In brief, RNA was decapped with tobacco decapping enzyme and randomly fragmented to approximately 200 nucleotides using RNA fragmentation reagents. Protein A/G beads were conjugated with an m7G antibody by incubation at room temperature for 1 h. Fragmented RNA was then incubated with the antibody-coupled beads at 4°C for 4 h. Following immunoprecipitation, RNA/antibody complexes were digested with proteinase K, and the eluted RNA was purified by phenol: chloroform extraction. RNA libraries for both IP and input samples were prepared using the NEBNext Ultra II Directional RNA Library Prep Kit (New England Biolabs, Inc., USA). Library quality was assessed on an Agilent 2100 Bioanalyzer, and paired-end sequencing was carried out on an Illumina NovaSeq platform.

Paired-end reads generated from the Illumina NovaSeq 6000 platform underwent quality control, with Q30 used as the quality threshold. Adapter sequences were trimmed, and low-quality reads were filtered out using Cutadapt (v1.9.3) to obtain high-quality clean reads. Clean reads from input libraries were first aligned to the UCSC HG19 reference genome using STAR software. Subsequently, DCC software was applied to identify mRNAs based on the STAR alignment results. Following this, clean reads from all libraries were aligned to the reference genome using Hisat2 (v2.0.4). Methylated sites (peaks) on mRNAs were detected with MACS software, and differentially methylated sites were identified using diffRep. Finally, peaks identified by both tools that overlapped with exonic regions of protein-coding genes were selected and extracted using custom scripts.

To identify biologically relevant motifs, sequence analysis of methylation peaks was conducted using DREME software. In our DREME analysis, we applied an E-value threshold of < 0.05 for reporting significantly enriched motifs. The significance of each motif was evaluated based on its E-value, which is calculated as the product of the enrichment p-value and the total number of candidate motifs tested. The enrichment p-value was derived using Fisher’s exact test to assess motif overrepresentation in the target sequences. Notably, motif credibility is inversely related to the E-value, meaning a lower E-value corresponds to higher confidence in the motif. Additionally, Gene Ontology (GO) analysis and KEGG pathway enrichment analysis were performed to elucidate the biological functions and pathways associated with genes containing differentially methylated mRNAs.

### Transcriptome sequencing and statistical analysis

High-quality sequencing reads were aligned to the mouse reference genome using HISAT2 software (version 2.0.4). Guided by the Ensembl GTF gene annotation file, Cuffdiff software (version 2.2.1, part of Cufflinks) was employed to calculate FPKM values as mRNA expression profiles. Differentially expressed mRNAs were identified based on fold change and p-value derived from FPKM data. We followed the strategy employed by https://doi.org/10.1371/journal.pone.0125722 [11], adopting a combined threshold of fold change≥2, p-value≤0.05 and FPKM ≥ 0.5 (in at least one sample), ensuring that differential expression calls are grounded in genuine transcriptional signals. Statistical analyses were performed using SPSS 25.0 and GraphPad Prism 10.0. Differential expression/peaks were identified using the edgeR exact test based on the negative binomial distribution/ diffReps negative binomial test.

## Results

**Fig 1:**
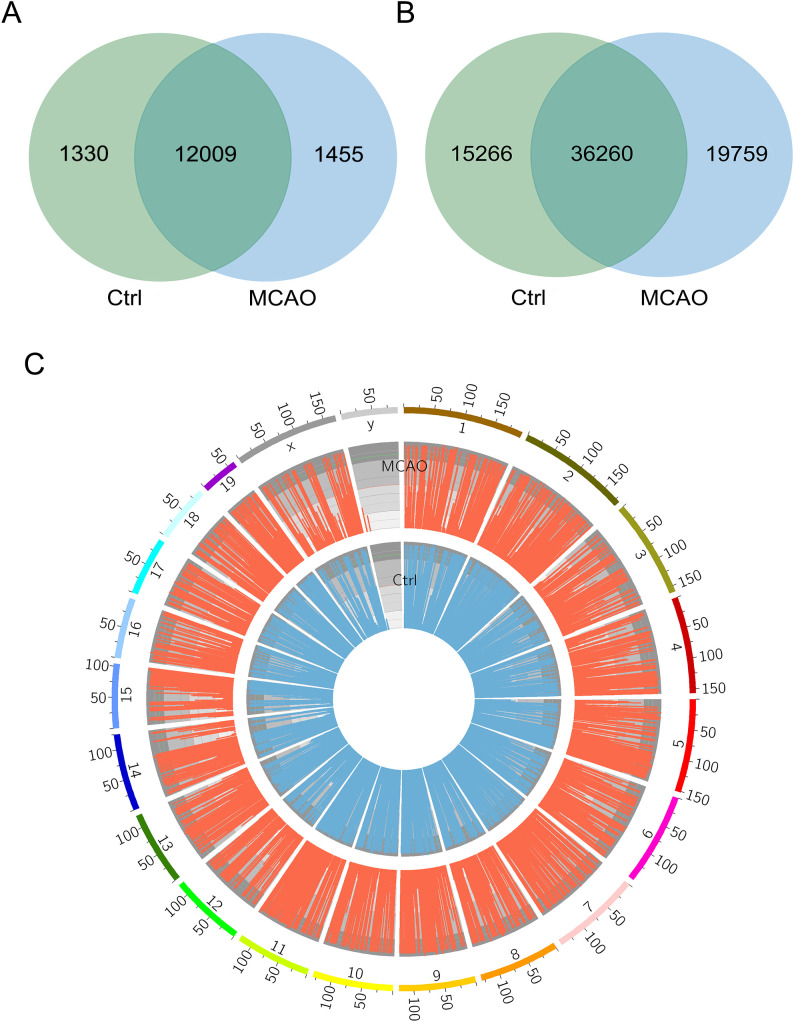
Overview of m7G methylation in Control and MCAO group. **(A)** Venn diagram of m7G genes in Control and MCAO group. **(B)** Venn diagram of m7G peaks in Control and MCAO group. **(C)** Visualization of m7G at the chromosome level in Control and MCAO group. The number of m7G peaks on chromosomes 1 and 2 was the highest and the number of m7G peaks on chromosome 19 was the lowest.

Transcriptome-wide m7G methylation sequencing and RNA sequencing were conducted in both the Control and MCAO groups. In the Control group, 51,526 high-quality methylation peaks were detected, corresponding to transcripts from 13,339 genes. In contrast, the MCAO group exhibited 56,019 peaks associated with 13,464 gene transcripts. Analysis revealed that 36,260 peaks were common to both groups. Compared to the MCAO group, the Control group contained 15,266 unique peaks and lacked 19,759 peaks present in the MCAO group, demonstrating significant differences in m7G methylation patterns between the two groups (Fig 1A, B).

### Distribution characteristics of m7G methylation

Analysis of mRNA m7G peak distribution across chromosomes revealed variations in both the quantity and localization of peaks on each chromosome. Chromosomes 1 and 2 exhibited the highest number of m7G peaks, while chromosome 19 displayed the lowest. Furthermore, autosomes generally demonstrated higher levels of methylation compared to sex chromosomes in both experimental groups (Fig 1C).

### Analysis of differentially regulated m7G methylated genes

To examine differential expression of m7G-methylated genes between the Control and MCAO groups, we statistically analyzed the m7G-modified genes from both conditions. Genes with a methylation fold change greater than two and a p-value ≤ 0.00001 were classified as significantly upregulated or downregulated. Relative to the Control group, MCAO penumbra tissue exhibited upregulated m7G methylation in 1,323 genes and downregulated methylation in 1,770 genes. The top 15 genes showing the most pronounced upregulation and downregulation are detailed in Tables 1 and 2, respectively.

**Table 1:** Top 15 up-methylated genes.

Chromosome	txStart	txEnd	Gene name	Fold change
chr7	126551945	126552245	Sez6l2	13374.6
chr4	118994021	118994440	Slc2a1	12375.8
chr2	61641926	61643180	Tbr1	11012.9
chr7	3415241	3415640	Cacng7	8797
chr8	9257481	9257980	Fam155a	7186.3
chr19	45780961	45781340	Kcnip2	5971.7
chr4	118993481	118993820	Slc2a1	4721.9
chr7	3416221	3416580	Cacng7	4526.4
chr7	126549734	126550080	Sez6l2	4144.85
chr4	125005461	125005940	Meaf6	3534.9
chr16	4895221	4895720	Ubn1	3515.4
chr6	108526713	108526800	Itpr1	3491.6
chr5	115784221	115784580	Rab35	3363.6
chr16	17610289	17610640	Klhl22	3153.7
chr17	49403721	49404340	Lrfn2	3072.6

**Table 2:** Top 15 down-methylated genes.

Chromosome	txStart	txEnd	Gene name	Fold change
chr14	55344541	55344980	Jph4	9327.2
chr14	55345021	55345220	Jph4	7473.9
chr19	42063521	42064060	Morn4	3804
chr6	148139021	148139380	Tmtc1	2897.9
chr17	44264005	44264380	Rcan2	2804.6
chr9	45061708	45062100	Scn4b	2696
chr9	45062901	45063340	Scn4b	2469.8
chr11	115144523	115144753	Grin2c	2201.6
chr4	152200799	152201180	Tnfrsf25	2089.4
chr4	155353489	155353609	Prkcz	1799.2
chr6	124441541	124441753	Clstn3	1621.1
chr19	42064161	42064420	Morn4	1469.8
chr9	59542138	59542264	Parp6	1429.2
chr5	24361901	24362089	Klhl7	1306.7
chr1	36510721	36511520	Cnnm4	1284.5

### Motif analysis of m7G methylation

**Fig 2:**
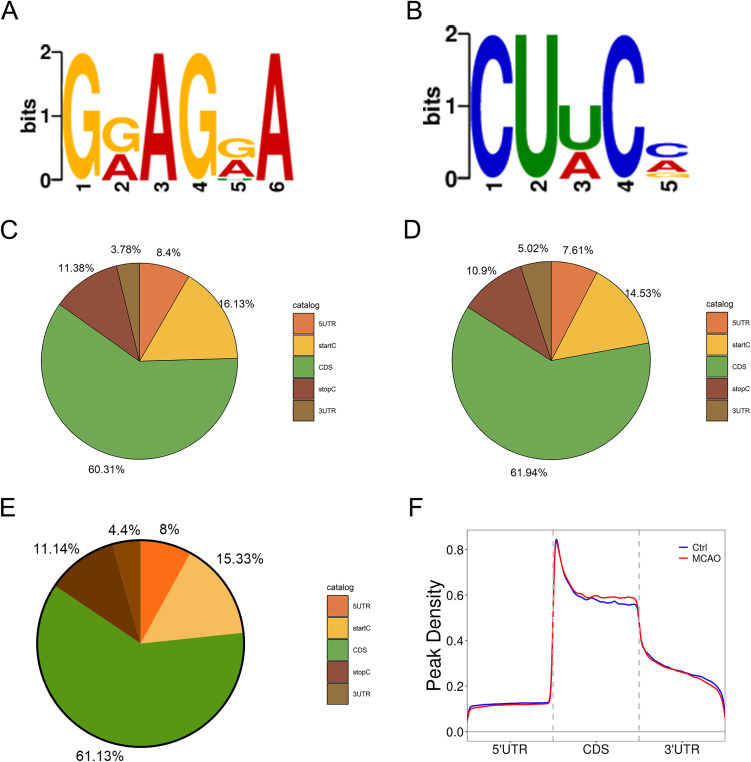
Motif and regional analysis of m7G methylation. **(A)** Motif with minimum E-value of m7G in Control group. **(B)** Motif with minimum E-value of m7G in MCAO group. **(C)** Pie chart of m7G peaks in different regions of mRNA in Control group. **(D)** Pie chart of m7G peaks in different regions of mRNA in MCAO group. **(E)** Pie chart of m7G peaks in different regions of mRNA in differentially modified genes of the two groups. **(F)** Analysis of the peak density modified by m7G methylation.

In this study, DREME software was employed to identify conserved motif sequences. The most significant motifs identified were GRAGDA (where R = G/A, D = G/A) and CUWCV (where W = U/C, V = C/A), with E-values of 1.8e-248 in the Control group and 7.7e-235 in the MCAO group, respectively (Fig 2A, 2B). Notably, the motif sequences differed markedly between the two groups.

### Regional analysis of m7G methylation

The analysis of m7G methylation sites revealed that this modification is distributed across all mRNA regions. The highest density of m7G methylation was observed in the CDS region, followed by the StartC region, while the StopC and 5’UTR regions showed the lowest methylation levels (Fig 2C, D). The regional distribution pattern of differentially methylated genes was similar between the Control and MCAO groups (Fig 2E). Furthermore, statistical analysis of m7G peak density demonstrated comparable numbers of m7G peaks in the CDS region in both groups. Compared to MCAO penumbra tissue, the Control group exhibited a similar number of m7G peaks in the 5’UTR region, but a greater number in the 3’UTR region (Fig 2F).

### Integrated analysis of differentially expressed m7G-methylated genes and mRNA expression profiles

**Fig 3:**
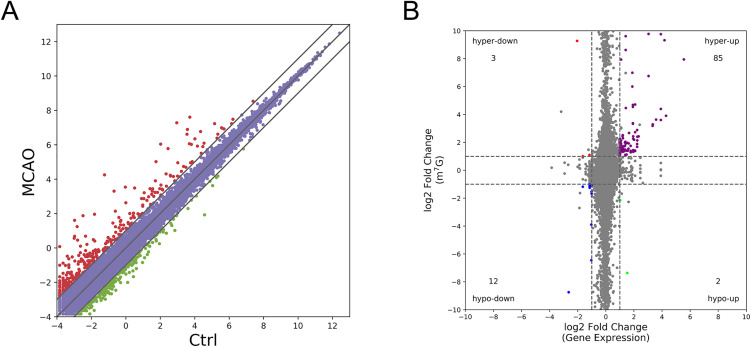
Joint analysis of m7G methylation and transcriptome. **(A)** Scatter plots of the differentially expressed mRNAs between Control and MCAO group. **(B)** Conjoint analysis of m7G methylation and the transcriptome of mRNA expression.

RNA sequencing analysis identified 3,093 genes with statistically significant expression differences in the MCAO group compared to the Control group, including 1,323 upregulated and 1,770 downregulated genes. Differentially expressed mRNAs were visualized using scatter plots (Fig 3A). Integrated analysis of m7G methylation and mRNA expression revealed distinct patterns of epigenetic regulation. Among the 14 genes with low mRNA expression, m7G methylation was significantly upregulated in two genes and significantly downregulated in 12 genes (fold change > 2, p < 0.001), classified as ‘hypo-up’ and ‘hypo-down’, respectively. Conversely, among the 88 genes with high mRNA expression, 85 genes showed significant upregulation and three genes showed significant downregulation in m7G methylation, categorized as ‘hyper-up’ and ‘hyper-down’ (Fig 3B).

### Bioinformatic analysis of m7G-methylated genes

**Fig 4:**
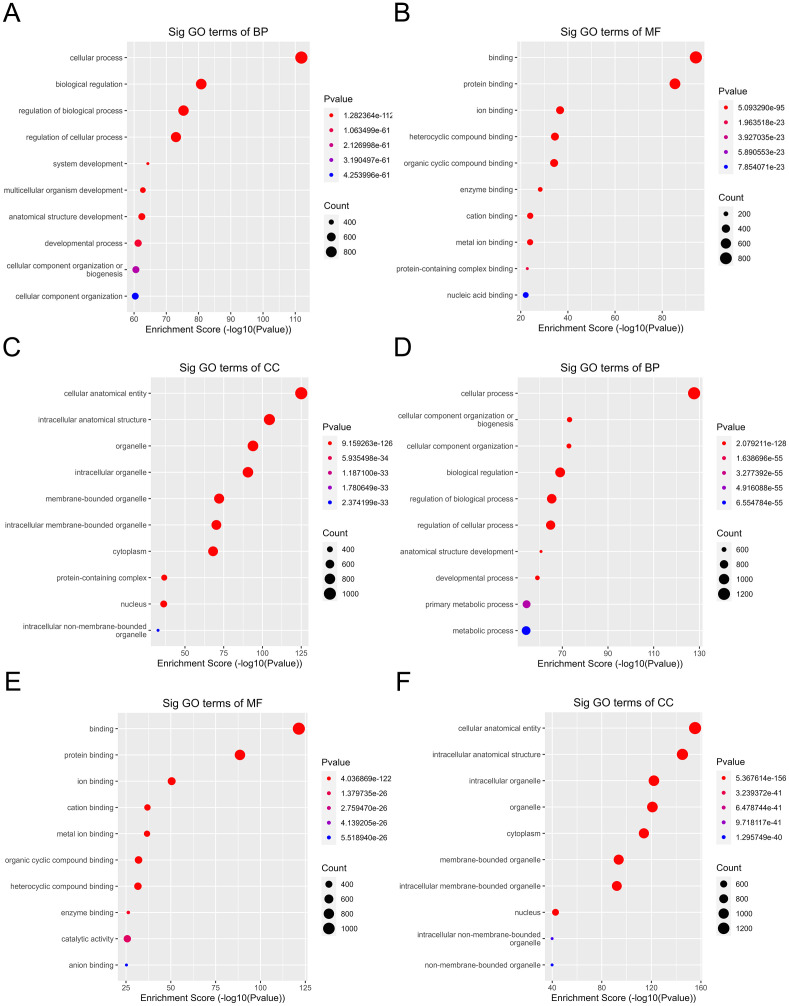
GO analysis on different m7G methylation genes of Control and MCAO group. **(A-C)** GO enrichment analysis of up-methylated m7G genes in MCAO group. **(D-F)** GO enrichment analysis of down-methylated m7G genes in MCAO group. GO, gene ontology; BP, biological process; CC, cellular component; MF, molecular function..

To elucidate the pathophysiological role of m7G methylation modifications in MCAO penumbra tissue, we performed Gene Ontology (GO) enrichment and KEGG pathway analyses on differentially methylated m7G genes in the MCAO group relative to the Control group. As shown in Fig 4, within the biological process (BP) category, m7G hypermethylated genes were predominantly associated with biological regulation, regulation of biological processes, and regulation of cellular processes, while m7G hypomethylated genes were primarily linked to cellular component organization or biogenesis, cellular component organization, and biological regulation. In the molecular function (MF) category, hypermethylated genes were enriched for terms such as binding, protein binding, ion binding, heterocyclic compound binding, and organic cyclic compound binding, whereas hypomethylated genes mainly involved binding, protein binding, ion binding, cation binding, and metal ion binding. Regarding cellular components (CC), hypermethylated genes were largely related to cellular anatomical entity, intracellular anatomical structure, and organelle, while hypomethylated genes were significantly associated with cellular anatomical entity, intracellular anatomical structure, intracellular organelle, and organelle.

**Fig 5:**
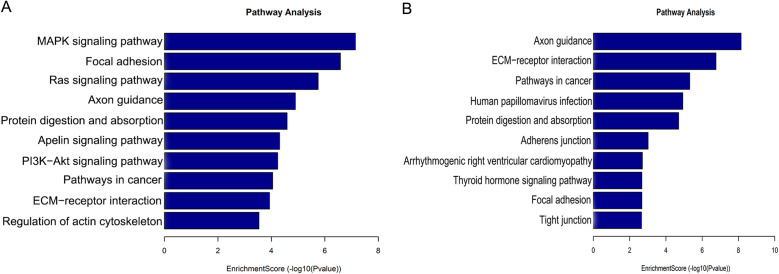
KEGG analysis on different m7G methylation genes of Control and MCAO group. **(A)** Major enriched KEGG pathway of up-methylated m7G in MCAO group. **(B)** Major enriched KEGG pathway of down-methylated m7G in MCAO group.

KEGG pathway analysis indicated that mRNAs with upregulated m7G methylation in the MCAO group were predominantly enriched in the MAPK signaling pathway, Focal adhesion, and the Ras signaling pathway (Fig 5A). Conversely, mRNAs exhibiting downregulated m7G methylation were significantly associated with Axon guidance, ECM receptor interaction, and Pathways in cancer (Fig 5B). The top ten most significantly enriched terms in each category are presented in Figs 4 and 5.

## Discussion

Epigenetic gene modifications represent a prominent area of research in brain development and neurological disorders. Emerging evidence indicates that dysregulated epigenetic mechanisms, including RNA methylation [12], DNA methylation [13], histone covalent modifications [14], and non-coding RNAs [15], play critical roles in both normal brain development and the pathogenesis of neurological diseases.

Current research has demonstrated an association between specific gene methylation events and the onset and progression of stroke. Detecting these methylation changes is also significant for prognostic assessment. Recent studies highlight the crucial role of m6A modifications in the pathophysiology of ischemic stroke [16]. Multiple lines of evidence indicate that aberrant m6A methylation contributes to key ischemic cascade events, including oxidative stress, apoptosis, neurogenesis, mitochondrial dysfunction, glutamate-mediated excitotoxicity, and inflammatory responses [17].

The role of N7-methylguanosine (m7G) methylation has recently garnered growing interest in neurological research. Multiple RNA-seq studies of mouse brain tissue following ischemic stroke have shown altered expression of METTL1 mRNA after middle cerebral artery occlusion (MCAO) [18]. Notably, levels of let-7e-5p, a microRNA whose transcripts are a validated target of METTL1, were significantly elevated in serum and cerebrospinal fluid from patients with ischemic stroke, consistent with increased METTL1 expression observed in MCAO mouse brains [19]. Despite these associations, research specifically focused on mRNA m7G methylation modifications in ischemic stroke remains limited.

In this study, we performed MeRIP-seq to profile mRNA m7G methylation peaks in both control and middle cerebral artery occlusion (MCAO) penumbra tissues. Comparative analysis revealed over 500,000 m7G peaks and nearly 100,000 m7G-methylated genes, with significant differences in m7G modification patterns between the two groups. While 81% of modified genes and 51% of modification peaks were shared between conditions, the overall m7G methylation level was higher in MCAO penumbra tissue compared to controls. These findings suggest that m7G methylation may contribute to the molecular mechanisms underlying ischemic penumbra pathophysiology. MeRIP-seq, as the most widely adopted method for transcriptome-wide RNA methylation profiling, has provided critical technical support for delineating the m7G methylome in this study. Nonetheless, we are acutely aware that this method has several inherent technical biases and limitations, with antibody non-specific binding representing the primary source of bias. To minimize the impact of this bias on our conclusions, we implemented the following measures: (1) For each biological sample in the MeRIP-seq experiment, we simultaneously sequenced both the immunoprecipitated (IP) library and the Input control library, and defined methylated regions strictly as those where the IP signal was significantly higher than the Input signal; (2) For the key differentially methylated genes identified, we performed independent validation using MeRIP-qPCR, thereby corroborating the robustness of the bioinformatic findings.

Epigenetic modification represents a critical area of investigation in ischemic stroke pathogenesis. Recent studies have established that dysregulated epigenetic mechanisms, including RNA methylation, DNA methylation, histone modifications, and miRNA aberrations, contribute significantly to the onset and progression of ischemic stroke [20]. Altered methylation of specific genes has been linked to disease development, and detecting these modifications holds prognostic value. For instance, the glucose transporter GLUT1 (Slc2a1), expressed in endothelial cells and astrocytes, plays a key role in brain energy metabolism [21,22]. Enhanced astrocytic glucose metabolism following GLUT1 reduction has been associated with neuroprotection in stroke models [23]. In our study, Slc2a1 exhibited significant upregulation in m7G methylation in the MCAO group. Ischemic stroke and reperfusion impair molecules regulating calcium homeostasis, such as Itpr1, which typically shows reduced expression post-stroke [24]. Interestingly, our data revealed that Itpr1 was significantly upregulated in m7G methylation in the MCAO group. Among genes with downregulated methylation, Tmtc1, a regulator of vascular responses linked to intracranial atherosclerotic stenosis (ICAS), may serve as a potential diagnostic and prognostic marker for ICAS with acute cerebral infarction [25]. Additionally, genes including Scn4b (a susceptibility gene for atrial fibrillation) [26], Prkcz (associated with acute coronary syndrome) [27], and CircAFF2 (implicated in neuronal injury via the miR-488/CLSTN3 axis) [28]were also identified, underscoring the complex molecular network influenced by m7G methylation. Despite these associations, the precise role of m7G methylation in ischemic stroke remains unclear. Our motif analysis revealed distinct m7G methylation patterns between control and MCAO groups, suggesting that differential methylation may be driven by variations in m7G methyltransferase activity or specificity. However, further experimental validation is required to elucidate the underlying mechanisms and causal relationships.

Numerous studies indicate that the regional distribution of methylation sites within mRNA is critical for regulating mRNA stability and translation. Meyer et al. reported that m6A modifications are enriched near stop codons and within 3’UTRs, where they can influence microRNA binding and thereby exert post-transcriptional regulatory effects [29]. Another recent study demonstrated that increased m6A methylation in the coding sequence (CDS) region of HSPA1A enhances its mRNA stability, leading to elevated HSPA1A protein expression [30]. In our study, we observed that m7G methylation was predominantly located in the CDS region in both control and MCAO groups, and the majority of differential methylation between the two groups also occurred within the CDS. Whether m7G methylation similarly modulates mRNA stability and expression in ischemic stroke penumbra tissue remains to be confirmed and warrants further experimental investigation.

The axon guidance pathway is essential for neural repair and post-stroke plasticity. Stroke disrupts cerebral connectivity, and the reactivation of axon guidance molecules, such as Netrin-1, Slit2, Sema3A, Sema4D, Ephrin-B2, and EphA4, can facilitate recovery by promoting axonal sprouting, rewiring, and synaptic reorganization. These molecules exert diverse effects: Netrin-1 enhances neuroprotection and axonal regeneration [31,32]; Slit2 may encourage axonal sprouting in peri-infarct regions [33]; Sema3A can redirect growing axons [34,35]; Sema4D promotes angiogenesis and neuroprotection [36,37]; Ephrin-B2 supports vascular remodeling and axonal sprouting [38]; and inhibition of EphA4 reduces glial scar formation, thereby improving recover [39]. Nevertheless, the influence of RNA methylation on the axon guidance pathway remains poorly understood. Notably, our KEGG analysis revealed significant downregulation of m7G methylation in axon guidance-related genes within ischemic stroke penumbra tissue. This suggests that reduced m7G methylation may positively regulate axon guidance genes, contributing to molecular adaptations after stroke. Conversely, the MAPK signaling pathway plays a critical role in stroke pathophysiology, modulating neuronal survival, inflammation, apoptosis, and recovery processes. Following a stroke, MAPK pathways, including ERK, JNK, and p38, are activated in response to ischemia-reperfusion injury, oxidative stress, and inflammatory signals. While the ERK pathway generally promotes cell survival and neuroprotection [40,41], JNK and p38 pathways are often associated with apoptosis and inflammation [42,43]. How RNA methylation affects MAPK signaling in stroke remains unclear. In our study, KEGG analysis showed significant upregulation of m7G methylation in MAPK pathway-related genes in the ischemic penumbra. This study represents the first exploration of the molecular regulatory role of m7G methylation in the ischemic penumbra during acute ischemic stroke. Our findings provide novel insights into epitranscriptomic mechanisms underlying penumbral progression and highlight potential targets for early therapeutic intervention, offering promising strategies to salvage at-risk tissue and improve clinical outcomes.

### Study limitations

One limitation of this study is its relatively small sample size. Therefore, further research involving larger cohorts is necessary to validate and extend these findings. The exclusive use of young male BALB/c mice in this study indeed limits the generalizability of our findings to broader populations, particularly females and aged individuals.

## Abbreviations

AIS: Acute ischemic stroke
MAPK: Mitogen-activated protein kinase
PEN: Pmethyltransferase-like 1
IACUC: Institutional Animal Care and Use Committee
MCAO: Middle cerebral artery occlusion
GO: Gene Ontology
BP: Biological process
MF: Molecular function
CC: Cellular components
ICAS: Intracranial atherosclerotic stenosis
CDS: Coding sequence
