# Supplementary material for: A novel m7G RNA methylation-related signature associated with MAPK signaling pathways in acute ischemic stroke
Source: PLoS One. 2026 Jun 23;21(6):e0352280. doi: 10.1371/journal.pone.0352280 (PMC13289932; doi:10.1371/journal.pone.0352280)
Supplement: Attachment — Submitted filename: Response to Reviewers.docx [file pone.0352280.s002.docx]

Dear Chandrabose Selvaraj：

Thank you for giving us the opportunity to submit a revised draft of our manuscript titled 'A Novel m7G RNA Methylation-related Signature Associated with MAPK Signaling Pathways in Acute Ischemic Stroke' (Manuscript ID: PONE-D-26-11064) to PLOS ONE. We appreciate the time and effort that you and the reviewers have dedicated to providing your valuable feedback on our manuscript. We are grateful to the reviewers for their insightful comments on our paper.

We have been able to incorporate changes to reflect most of the suggestions provided by the reviewers. We have highlighted the changes within the manuscript using Track Changes. Here is a point-by-point response to the reviewers' comments and concerns. All page numbers refer to the revised manuscript file with tracked changes.

Thank you again for your consideration.

Sincerely yours,

Wu Wei PhD.

Reviewer #1: The research work is considered absolutely novel. However, the in vivo methodology is insufficiently detailed. The following concerns need to be addressed.

1. There are four concerns regarding the statement: “Focal cerebral ischemia was induced using a silicone-coated nylon filament inserted into the middle cerebral artery (MCA) for 2 h [9]”.

First, the filament is typically advanced through the internal carotid artery (ICA) until it reaches the origin of the MCA to effectively block blood supply to the MCA territory. Therefore, the “silicone-coated nylon filament inserted into MCA” statement needs to be revised appropriately.

Second, the authors should specify the filament size/number and the supplier details.

Third, in young mice, ischemia is commonly induced for 1 hour. The authors should justify the use of a 2-hour occlusion duration, which is more frequently employed in rat models. Notably, even 1 hour of ischemia in mice can significantly increase mortality rates.

Fourth, Reference 9 (Goodman et al., 2023) reports using 30–90 minutes of ischemia to induce stroke, whereas the authors have used a 2-hour duration. Considering that BALB/c mice are generally more susceptible to cerebral ischemic injury and exhibit higher mortality rates (up to 71%–100%) in MCAO models compared to C57BL/6 mice, a 2-hour occlusion represents a severe insult and is likely associated with substantial mortality. Any mortality observed in the study should therefore be clearly reported.

Response: Thank you for pointing this out. We have re-described the steps of the MCAO ischemia model procedure on page 6, line 21 - page 7, line 4 of the revised manuscript. We have supplemented the specifications/model and supplier information of the filament in the Methods section on page 6, line 21 of the revised manuscript. We did not clearly describe the duration of ischemia induction in the MCAO model. The ischemia induction time in mice was 1 hour, and euthanasia was performed 2 hours after successful induction of the ischemic model on page 7, line 2-3 of the revised manuscript.

2. The authors should clarify whether the study complies with ARRIVE guidelines. In accordance with these guidelines, the authors are expected to report the inclusion and exclusion criteria used to confirm stroke induction in the animals, any exclusions made, details of the experimental groups in the methods, the sample size for each group, post-surgical care provided to the animals, and the source/vendor of the animals.

Response: We thank the reviewer for this valuable comment. We confirm that our study has been conducted and reported in accordance with the ARRIVE guidelines (Animal Research: Reporting of In Vivo Experiments). Accordingly, we have provided the following information in the revised manuscript (page 7, lines 14-22):

This study complies with the ARRIVE guidelines (Animal Research: Reporting of In Vivo Experiments). Inclusion criteria were a reduction in cerebral blood flow (CBF) of ≥70% as measured by laser Doppler flowmetry and a neurological behavior score of ≥1. Exclusion criteria included intraoperative death, absence of significant CBF reduction, or the presence of subarachnoid hemorrhage. No animals died during the modeling procedure. After surgery, the animals were placed in temperature-controlled cages (37 ± 0.5°C) until they regained consciousness. They were housed individually and provided with moistened chow and hydrogel for hydration. Neurological behavior and the condition of the surgical incision were monitored. Analgesic treatment was administered in accordance with the regulations on laboratory animal welfare.

3. It is stated that the animals were euthanized; however, the specific time point of euthanasia is unclear (e.g., Day 1, Day 2, or Day 3).

Response: We agree with the reviewer's assessment. We did not clearly describe in our manuscript that the time point for euthanizing the experimental animals was 2 hours after successful MCAO model establishment. This change can be found on page 7, lines 2-3 of the revised manuscript.

4. No details are provided regarding “saline or PBS transcardiac perfusion” or the “snap freezing” of brain tissues collected for transcriptomic sequencing analysis. The authors should clarify whether these procedures were performed.

Response: Thank you for this valuable suggestion. Following your suggestion, we have added the relevant experimental procedures in the Methods section on page 7, lines 7-13 of the revised manuscript:

The tissue was then rinsed in pre-chilled PBS or saline to remove blood stains and debris. The target ischemic penumbra tissue was rapidly dissected on ice. Using forceps, the tissue block was picked up and directly immersed into liquid nitrogen, ensuring that the tissue was completely submerged. After freezing for several seconds until the tissue became completely hardened, the snap-frozen tissue block was removed and quickly transferred into a pre-chilled, labeled cryotube. The cryotube was then stored in a -80°C freezer for long-term preservation.

5. In the statistical analysis, it is unclear whether a paired or unpaired Student’s t-test was used. Additionally, it is not specified whether a normality test was performed prior to applying the t-test to confirm that the data follow a Gaussian distribution.

Response: We sincerely appreciate your insightful comments, which are essential for enhancing the statistical rigor of our manuscript. We would like to address your concerns regarding the statistical tests as follows:

Regarding the type of t-test (paired or unpaired) and normality testing

First, we apologize for any ambiguous wording in the initial Methods section. In our actual analytical pipeline, we did not employ a conventional Student's t-test for either the MeRIP-seq or RNA-seq data analysis. Consequently, no normality test was performed as it is not a prerequisite for the models used.

Clarification of the actual statistical models applied

MeRIP-seq Analysis (using diffReps): Given that MeRIP-seq data consist of discrete read counts, they inherently exhibit overdispersion (variance increases with the mean) and do not follow a Gaussian distribution. Therefore, we utilized the Exact Negative Binomial Test, which is the recommended default method in diffReps (v1.55.6) for data with biological replicates. This model specifically accounts for count data properties and does not assume normality.

RNA-seq Analysis (using edgeR): Also based on the characteristics of count data, edgeR (v3.16.5) uses empirical Bayes estimation and exact tests based on a negative binomial model to identify differentially expressed genes. This framework does not assume that the data follow a normal distribution, so the workflow does not include a normality test step.

Manuscript Revision

To prevent any future misunderstanding, we have revised the statistical analysis subsection of the Methods to explicitly state: "Differential expression/peaks were identified using the edgeR exact test based on the negative binomial distribution / diffReps negative binomial test." We thank you again for your thorough review and the opportunity to clarify this important methodological detail (page 10, lines 7-8).

6. In Figures 1A and 1B, the font size of the values in the Venn diagram should be increased to improve visibility.

Response: Thank you for pointing this out. We have increased the font size of the numbers within the Venn diagrams in Figures 1A and 1B to improve readability. See Figure 1 for details.

7. In Figure 5, the font size of the labels on the Y-axis should be increased for better readability

Response: Thank you for pointing this out. We have increased the font size of the Y-axis labels in Figure 5 to improve readability. See Figure 5 for details.

Reviewer #2:

1. Why only deploy small sample size of 12 mice. It could potentially reduce statistical power and increase the chance of errors. The generalizability of the finding could be weaken as well.

The current sample size should be justified for observing subtle but biologically relevant differences in m7G methylation or gene expression may go undetected.

Response: Thank you for raising this important point. For transcriptome sequencing (RNA-seq) studies, particularly when using a model animal such as the mouse, a sample size of n=6 per group can, to some extent, balance statistical power and cost. This sample size is sufficient to detect moderate to large differences in gene expression. Based on the characteristics of RNA-seq data (negative binomial distribution), with n=6 per group, for genes with a fold change ≥ 2 and moderate expression levels, the statistical power typically exceeds 80% after correction for multiple hypothesis testing (e.g., FDR < 0.05). A sample size of 8–12 per group can significantly enhance the ability to fully replicate experimental results, achieving a good balance between controlling false positives and maintaining sensitivity. Our study does have a relatively small sample size, and we have addressed this limitation in the Study Limitations. Furthermore, we plan to validate the identified differentially expressed genes in a larger sample set in future studies.

2. There is a limited scope of animal model, because only young male BALB/c mice were deployed. It important to consider this matter because sex could play part in the stroke outcomes. For instance, hormonal difference in female mice could potentially affect the outcomes.

Response: We thank the reviewer for raising this important limitation. We fully agree that the exclusive use of young male BALB/c mice limits the generalizability of our findings, especially given the known influence of sex on stroke outcomes. The selection of young male mice in this study was based on the following considerations: 1) Variable control: To preliminarily establish the molecular mechanisms of this MCAO model, we first excluded potential confounding variables associated with hormonal cycle fluctuations in females; 2) Under the same conditions, the cerebral infarct volume in female mice is significantly smaller than that in males. To exclude the interference of estrogen and ensure the stability and reproducibility of experimental data, our conclusions were validated only in young male BALB/c mice. Meanwhile, we have addressed the limitation regarding the sex of the mice in the Study Limitations (page 18, lines 9-11). Future studies will include animals of both sexes and different age groups to comprehensively evaluate the molecular mechanisms of acute ischemic stroke.

3. For the DREME software, What is the E-value threshold that you used? Kindly provide the threshold to avoid both false positives and negatives!

Response: Thank you for your careful attention to the motif analysis parameters. In our DREME analysis, we applied an E-value threshold of < 0.05 for reporting significantly enriched motifs. This is the default recommended threshold of the DREME software. We have updated the Methods section of the manuscript to explicitly include this threshold information (page 15, lines 11-12).

4. You need to soften the claim related the link between m7G methylation and MAPK pathway, becuase the activation is not causative.

Response: Thank you for this important correction. We fully agree that our data only support an association between m7G methylation and the MAPK pathway, rather than a direct causative activation. We have softened the language in the conclusion of the abstract in the manuscript (page 15, lines 18-19). Although our data show a correlation between m7G methylation levels and MAPK pathway activation, further mechanistic studies are still needed to determine whether this reflects a direct regulatory interaction.

5. Kindly discuss the potential biases in MeRIP-seq as they could confound the interpretation of the methylation pattern. If the binding is non specific, it could potentially lead to false-positive signals.

Response: Thank you for raising this important point. We fully agree that MeRIP-seq harbors potential biases, and a thorough discussion of these limitations is essential for the objective interpretation of methylation patterns. Accordingly, we have added relevant content to the Discussion section (page 23, lines 1-11), as summarized below:

MeRIP-seq, as the most widely adopted method for transcriptome-wide RNA methylation profiling, has provided critical technical support for delineating the m7G methylome in this study. Nonetheless, we are acutely aware that this method has several inherent technical biases and limitations, with antibody non-specific binding representing the primary source of bias. To minimize the impact of this bias on our conclusions, we implemented the following measures: (1) For each biological sample in the MeRIP-seq experiment, we simultaneously sequenced both the immunoprecipitated (IP) library and the Input control library, and defined methylated regions strictly as those where the IP signal was significantly higher than the Input signal; (2) For the key differentially methylated genes identified, we performed independent validation using MeRIP-qPCR, thereby corroborating the robustness of the bioinformatic findings.

6. Provide reference citations on the threshold value of the FPKM!

Response: Thank you for your comment regarding the FPKM threshold. We adopted FPKM ≥ 0.5 as an expression filter based on the following statistical consideration: When using only fold change ≥ 2 and p-value ≤ 0.05 as filtering criteria, genes with FPKM values approaching zero in both groups may yield spuriously large fold changes due to minor fluctuations in read counts, thereby introducing false-positive differentially expressed genes. To mitigate this bias, we followed the strategy employed by doi:10.1371/journal.pone.0125722, adopting a combined threshold of fold change≥2, p-value≤0.05 and FPKM ≥ 0.5 (in at least one sample), ensuring that differential expression calls are grounded in genuine transcriptional signals. We have added the relevant clarification and citation to the Methods section (page 10, lines 3-6). Thank you again for your insightful feedback.

7. The functional validation of this study is limited, because the functional consequence of the epigenetics modifications are not experimentally validated. Without extensive functional assays, it is uncertain to observe any pattern that affect the disease outcome

Response: We thank the reviewer for pointing out this critical limitation. We fully agree that the current manuscript primarily presents the transcriptomic landscape of m7G methylation in the ischemic penumbra and indeed lacks experimental validation of the functional consequences at specific modification sites.

It must be acknowledged that this study is positioned as an exploratory, hypothesis-generating investigation. As the first genome-wide profiling of m7G modification in the ischemic penumbra in this field, our core objective was to map the differential methylation landscape and provide a repository of candidate targets for subsequent functional studies. The KEGG and GO enrichment analyses of the differentially methylated genes in our study revealed functional associations of pathways such as axon guidance and MAPK signaling, which computationally support the potential link between methylation patterns and disease outcomes. Furthermore, previous studies have confirmed that METTL1, the key methyltransferase catalyzing m7G (N7-methylguanosine) methylation (doi: 10.1038/s41586-022-05566-4.), can influence critical processes such as cell proliferation, invasion, and differentiation by regulating the MAPK signaling pathway (doi: 10.1016/j.gene.2024.148234.). METTL1 exerts a protective role after ischemic stroke through catalyzing m7G modification (doi: 10.1016/j.prp.2026.156465). In our future studies, we will focus on the functional validation of METTL1, m7G methylation modification, and alterations in the MAPK signaling pathway in ischemic stroke to further elucidate their biological functions.
